# Early Assessment of Efficacy and Safety of Biologics in Pediatric Allergic Diseases: Preliminary Results from a Prospective Real-World Study

**DOI:** 10.3390/children11020170

**Published:** 2024-01-28

**Authors:** Lucia Caminiti, Francesca Galletta, Simone Foti Randazzese, Paolo Barraco, Stefano Passanisi, Antonella Gambadauro, Giuseppe Crisafulli, Mariella Valenzise, Sara Manti

**Affiliations:** Pediatric Unit, Department of Human Pathology in Adult and Developmental Age “Gaetano Barresi”, University of Messina, Street Consolare Valeria 1, 98124 Messina, Italy; lucycaminiti@yahoo.it (L.C.); francygall.92@gmail.com (F.G.); simofotir@gmail.com (S.F.R.); paolo.barraco@outlook.it (P.B.); gambadauroa92@gmail.com (A.G.); crisafullig@unime.it (G.C.); mvalenzise@unime.it (M.V.); sara.manti@unime.it (S.M.)

**Keywords:** atopic dermatitis, asthma, chronic urticaria, dupilumab, efficacy, omalizumab, safety

## Abstract

Background: Despite the increasing interest in biologics for the management of allergic diseases, sparse real-world data are still available in the pediatric population. This study aimed to evaluate the early real-life efficacy and safety of omalizumab for patients with moderate-to-severe asthma and chronic spontaneous urticaria (CSU), and Dupilumab for patients with moderate-to-severe atopic dermatitis (AD). Methods: A prospective study enrolling children aged 6–18 years was designed to assess the efficacy and safety of biologic drugs at 16 weeks of treatment (T1). The effectiveness was measured using validated questionnaires (ACQ-5 for asthma, UAS7 for CSU, and EASI score for AD). Secondary outcome measures included reductions in inhaled corticosteroid (ICS) dosages, asthma-related hospitalizations/exacerbations, and quality of life (QoL) indicators (iNRS, sNRS, DLQI/cDLQI) for CSU and AD. Safety was expressed according to the descriptions of adverse events provided by EMA and FDA. Results: The study cohort consisted of eighteen children (mean age 12.9 ± 3.4 years). The omalizumab treatment significantly reduced ACQ-5 and UAS7 scores (*p* = 0.002 and *p* < 0.001, respectively). In patients with asthma, decreased ICS dosage and hospitalization/exacerbation rates were observed. QoL parameters significantly improved in CSU and AD patients. No severe adverse events were reported for either treatment. Conclusions: Our findings validate omalizumab and dupilumab as effective and safe therapeutic options for managing moderate-to-severe allergic diseases in children and adolescents.

## 1. Introduction

In recent years, efforts have been devoted to improving the management of allergic diseases in children, such as asthma, chronic spontaneous urticaria (CSU), and atopic dermatitis (AD) [1]. Advances in molecular biology have favored the development of several targeted monoclonal antibodies against chronic immune-mediated allergic diseases [2]. Among these, omalizumab and dupilumab have been increasingly used for the treatment of atopic children and adolescents [2]. Omalizumab is a humanized monoclonal antibody that binds to the Fcε portion of immunoglobulin E (IgE), decreasing total IgE levels and preventing interaction with the low-affinity (FcεRII) and high-affinity (FcεRI) receptors of basophils and mast cells, downregulating their expression [3]. To date, omalizumab is approved by the Food and Drug Administration (FDA) for treating moderate-to-severe persistent asthma in patients aged > 6 years, chronic spontaneous urticaria (CSU) in patients aged > 12 years, and nasal polyps in children older than 18 years of age [4]. In addition, several studies are ongoing involving omalizumab as an add-on to oral immunotherapy (OIT) or as monotherapy in food allergy [5,6]. Dupilumab is a humanized monoclonal antibody targeting interleukin-4 receptor (IL-4R). The linkage with α-subunit of interleukin-4 receptor inhibits IL-4 and IL-13 signaling and downregulates the Th2 cells [7]. According to FDA, dupilumab is indicated for treating moderate-to-severe AD inadequately controlled by topical therapies and/or severe asthma in children aged > 6 years, severe eosinophilic asthma in patients older than 6 years, and nasal polyposis in adults. [8]. Several randomized clinical trials (RCTs) and post-marketing surveillance studies confirmed the efficacy and the safety of these monoclonal antibodies in children with allergic disease [2]. However, there is still a lack of robust real-world data [9,10,11]. The primary outcome of our study was to assess the efficacy of omalizumab and dupilumab after the initial 16 weeks of treatment in real-life settings among a cohort of pediatric patients suffering from moderate-to-severe asthma, CSU, and AD. Additionally, our study aimed to evaluate the safety profile of these biologics.

## 2. Materials and Methods

### 2.1. Study Design

A prospective single-center study was conducted from October 2020 to October 2022 at the Pediatric Unit of “G. Martino” Hospital, University of Messina, Messina, Italy. Omalizumab received approval by the European Medicines Agency (EMA) for the treatment of asthma in children aged > 6 years in 2009 and for CSU in adolescents aged >12 years in 2014. Dupilumab was approved in youths aged > 12 years in 2019 and in children > 6 years in 2020. 

Inclusion criteria were participants aged 6–18 years with the following conditions: a diagnosis of moderate-to-severe asthma that is relatively unresponsive to optimized treatment with high doses of inhaled corticosteroid-long-acting beta agonist (ICS-LABA) or requiring high doses of ICS-LABA [12]; a diagnosis of uncontrolled CSU, based on a weekly Urticaria Activity Score (UAS7) > 16, despite H1-antihistamines being administered up to 4 times a day [13]; or a diagnosis of uncontrolled moderate-to-severe AD according to the Eczema Area and Severity Index (EASI) with a score >16, despite treatment with emollients and topical steroids [14].

Exclusion criteria included the previous use of omalizumab or dupilumab, a history of allergic or systemic reactions to one of the components of the biologic drug, as well as other chronic conditions or poor treatment adherence. All patients included in the study and their parents were appropriately informed and signed an informed consent form. The study was conducted according to Good Clinical Practice and in accordance with the Declaration of Helsinki [15] and received approval by the local ethical Committee (n°99-22). 

### 2.2. Efficacy Assessment

The primary efficacy assessment of omalizumab and dupilumab involved evaluating disease activity using validated standardized questionnaires: the asthma control questionnaire-5 (ACQ-5) for asthma, the UAS7 for CSU, and the EASI score for AD. For omalizumab in asthma treatment, a secondary efficacy parameter included assessing the reduction in ICS dosage, along with decreases in exacerbations and hospitalizations. Furthermore, the secondary efficacy endpoint for omalizumab in CSU and dupilumab in AD aimed to evaluate the quality of life (QoL) using numerical rating scales for itch and sleep disorders (iNRS and sNRS, respectively) specifically for AD, as well as the Dermatology Life Quality Index (DLQI) or children’s version (cDLQI) based on age (6–16 years) for both CSU and AD. Anamnestic and clinical data, including body mass index (BMI), family history, comorbidities, and previous treatments, were collected. The evaluation of efficacy and safety parameters occurred at baseline (T0), representing assessment 16 weeks before initiating therapy, and after 16 weeks of treatment (T1). 

### 2.3. Safety Assessment

Adverse reactions were reported according to the descriptions provided by EMA and FDA [16,17]. During follow-up visits, patients were asked about any adverse reactions related to drug administration, which was recorded in dedicated medical reports.

### 2.4. Dosing

Omalizumab and dupilumab were administered via subcutaneous injections. For patients affected by moderate-to-severe asthma, the dose of omalizumab ranged between 150 to 600 mg every two or four weeks, depending on the baseline serum total IgE level and body weight [16]. For patients with CSU, the administered dose was 300 mg every four weeks [16]. Dupilumab dosing for patients with severe AD varied based on body weight and age. Regarding patients aged 12–17 years, an initial dose of 400 mg followed by a dose of 200 mg every 2 weeks was administered to patients weighing < 60 kg, or rather a dose of 600 mg followed by 300 mg for patients > 60 kg [17]. Conversely, for patients aged < 12 years, an initial dose of 300 mg followed by a maintenance dose of 300 mg every 4 weeks was administered for body weights < 60 kg. Also, a starting dose of 600 mg followed by 300 mg every 2 weeks was given to patients weighing ≥ 60 kg [17]. 

### 2.5. Statistical Analysis

The sample variables were characterized using descriptive statistics. Numerical data were expressed as mean ± standard deviation (SD), while categorical variables were presented as absolute frequencies and percentages. A post hoc analysis was performed to compare data between the baseline and the end of the study. Normal distribution in the sample was confirmed through the Kolmogorov–Smirnov test, allowing for a parametric approach in the analysis. Changes in numerical variables were assessed using a paired *t*-test. Differences in categorical data were evaluated by measuring the percentage of variation between the 16-week period pre-treatment and the subsequent 16-week biologics course. Statistical analyses were conducted using SPSS 22.0 (IBM, New York, NY, USA) for Windows package. A *p*-value smaller than 0.05 was considered statistically significant.

## 3. Results

### 3.1. Study Population

Eighteen patients (61% male), with a mean age of 12.8 ± 3.4 years, were recruited. The study population was divided into three groups: patients with moderate-to-severe asthma (*n* = 6; 33.3%), patients with severe CSU (*n* = 7; 39%), treated with omalizumab, and patients with moderate-to-severe AD (*n* = 5; 27.7%), treated with dupilumab. Demographical and clinical characteristics of the study population were described in Table 1.

### 3.2. Omalizumab in Moderate-to-Severe Asthma

**Figure 1:**
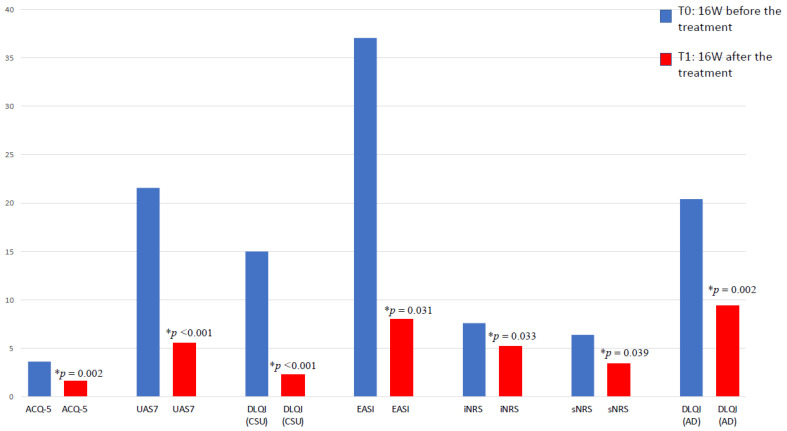
Changes in clinical scores 16 weeks before and after the treatment with biologics; * *p*-value < 0.005. ACQ-5: asthma control questionnaire-5; DLQI: Dermatology Life Quality Index; EASI: Eczema Area and Severity Index; iNRS/sNRS: numerical rating scales for itch and sleep disorders; UAS7: weekly Urticaria Activity Score.

Out of the six patients with asthma, 83.3% were males, with an average age at the start of the omalizumab of 10.5 ± 3.1 years. Five patients were also affected by allergic rhinitis, while four had allergic conjunctivitis [Table 1]. All analyzed patients exhibited a 55% reduction from baseline in ACQ-5 scores after 16 weeks (*p* = 0.002) [Figure 1]. Only one patient (16.6%) achieved complete symptom control with a score < 1. Although no patients discontinued ICS treatment, there was a reduction of 28.2% in ICS dosage, observed in only 50% (*n* = 3) of the cohort studied, which did not reach statistical significance. Similarly, a significant decrease in asthma exacerbations was highlighted in 76.93% of the patients. No hospitalization and/or Emergency Departments admissions were observed due to asthma exacerbations. However, these findings did not achieve statistical significance, likely due to the limited sample size.

### 3.3. Omalizumab in Severe CSU

Among the seven patients with CSU, 42.9% were male, starting the biologic drug at an average age of 13.9 ± 2.2 years. A significant reduction in UAS7 score of 74.8% after sixteen weeks was observed (*p* < 0.001). Patients showed a significant improvement in DLQI/CDLQI with a score reduction of 80% at 16 weeks (*p* < 0.001) [Figure 1].

### 3.4. Dupilumab in Moderate-to-Severe AD

Out of the five patients with AD, 60% were males, starting dupilumab at an average age of 14.4 ± 3.3 years. A reduction of 78.14% in EASI score (*p* = 0.031) was observed in the pediatric population that were treated with dupilumab in addition to medium potency topical corticosteroids (TCs). Approximately 60% of patients achieved the complete or almost complete resolution of skin lesions. Additionally, no extensions of the lichenification process were observed across the entire sample (100%). The study highlighted a decrease in iNRS and sNRS by 31.58% and 46.87%, respectively (*p* = 0.033 and *p* = 0.039, respectively). All patients met the target of reducing DLQI/CDLQI by at least 4 points, with an average reduction of 53.93% in the absolute score (*p* = 0.002). No exacerbations were reported.

### 3.5. Safety

AEs related to omalizumab administration were solely reported in asthma patients. Specifically, one patient (16.6%) described an isolated episode of acute urticaria, while two others (33.3%) experienced injection site pain. Conversely, patients undergoing omalizumab treatment for CSU did not report any AEs. On the other hand, among those receiving dupilumab, one patient (20%) reported erythema at the injection site, while two (40%) noted respiratory tract issues, which were likely unrelated to the drug administration. Notably, no severe AEs were reported during the follow-up period for either drug. After the end of the surveillance study, all patients continued treatment with biologics, which is still ongoing. 

## 4. Discussion

### 4.1. Efficacy and Safety of Omalizumab

Our study demonstrated the efficacy of omalizumab in treating moderate to severe asthma and CSU within the first 16 weeks of treatment. For asthma, regarding the primary efficacy endpoint, we recorded a significant reduction in the ACQ-5 score by 55.5% after 16 weeks. Only one patient experienced a complete symptom control with score reduction up to <1, confirming the literature findings. The 16-week time frame is crucial as it allows the assessment of the patients’ response to therapy, categorizing them as super-responders, partial responders, or non-responders [18]. A recent meta-analysis involving 565 patients with severe allergic asthma aged > 6 years showed a notable decrease in ACQ scores (MD [95% CI]: 1.14 [1.40 to 0.89]; I2 74%) on 16 weeks omalizumab therapy [19]. Furthermore, a reduction in ICS dosage was observed in 50% of our cohort, although no patients discontinued treatment. This outcome aligns with earlier findings. In a previous double-blind, placebo-controlled study conducted by Milgrom et al. [20] on 334 children with severe asthma aged 6–11 years, the omalizumab group showed a median ICS dosage reduction of 100% compared to 66.7% in the placebo group (*p* = 0.001). Also, 55% of the omalizumab cohort suspended ICS compared to 39% in the placebo group [19]. Recent studies have supported these findings [21,22,23]. An Italian real-life study of 47 patients revealed a reduction in ICS dosage in all subjects [24]. However, the initial ICS mean dose was lower, possibly due to a higher number of patients <12 years requiring low ICS doses [24]. Regarding asthma exacerbations, a significant reduction (76.93%) was noted in our cohort during omalizumab treatment, with no emergency department or hospital admissions due to asthma exacerbation. Although no new hospitalizations or emergency primary care admissions were observed in our study population, the statistical data on exacerbations and hospitalizations were not significant, likely due to the small sample size. Nevertheless, these findings were consistent with the previous literature data, which reported decreased annualized exacerbation rates in RCTs [25,26,27,28,29]. Moreover, real-life studies have consistently demonstrated a marked decrease in asthma exacerbations (up to 70–80%) within the initial six months of therapy, sustained over subsequent years [20,21,24,30]. Similar outcomes were observed for children aged 6–11 years and adolescents/adults [23]. A Cochrane meta-analysis of 25 trials, comprising 6382 patients treated with omalizumab for uncontrolled asthma, supported these findings by demonstrating decreased exacerbations, reduced hospitalizations, and increased ICS cessation or reduction [31]. Regarding the effectiveness of omalizumab in treating CSU, our study revealed a substantial reduction in exacerbations across the entire sample, marked by a 75% decrease in UAS7 scores from baseline to sixteen weeks. RCTs have demonstrated that current dosages of omalizumab induce a clinically significant decrease in UAS7 after 24 weeks with moderate certainty of evidence (MD −11.05; 95% CI −12.87 to −9.24) [32,33,34,35]. Furthermore, the achievement of a complete response (UAS7 = 0) was also high (95% CI 3.05 to 7.37) [35]. Real-world studies have consistently supported these findings [36,37]. A recent meta-analysis of real-life evidence, including 15 studies enrolling 294 patients with CSU, showed a substantial improvement in UAS7 scores (−25.6 points, 95% CI, −28.2 to −23.0; *p* < 0.001) [37]. In our study, another efficacy indicator considered was improved patient quality of life, measured via DLQI/cDLQI. We observed an 80% reduction in DLQI/CDLQI scores. This outcome aligns with findings from both RCTs [33,34,38] and real-life studies [37]. A randomized study conducted by Casale et al. [39], involving both adults and adolescents, assessed DLQI worsening of ≥3 points after treatment discontinuation. The placebo group showed a greater likelihood of DLQI worsening when compared to the omalizumab group (RR 3.34; 95% CI 2.07 to 5.40) [39]. Regarding the safety profile, we observed an isolated case of acute urticaria in an asthmatic patient two weeks after starting the medication, which spontaneously resolved and did not reoccur. Two cases reported immediate AEs of pain at the injection site, also resolving spontaneously within a few hours. No case of anaphylaxis and/or other allergic reactions were registered. According to the drug manufacturers, administration site reactions occurred in 45% of cases in adults and adolescents, compared to 43% in patients receiving a placebo [4]. In a double-blind, placebo-controlled study, skin rashes and urticaria were mild, with a similar prevalence in both groups [20]. Di Bona et al. [40] reported only one patient with urticaria and angioedema who had similar episodes before starting omalizumab. Thus, various randomized clinical trials (RCTs) and post-marketing surveillance studies suggest that omalizumab is generally safe and well-tolerated, with very low frequencies and severity of adverse events (AEs) in both short-term and long-term periods [41,42,43]. Real-life studies showed a lower incidence rate of AEs compared to RCTs [24,43,44,45]. In a recent prospective real-life study conducted by our group, evaluating the long-term safety of omalizumab in patients aged 6–18 years with asthma and/or CSU, no cases of anaphylaxis and/or other severe AEs were reported after 4 years of treatment [46]. Nonetheless, further studies with longer observation periods are necessary to comprehensively evaluate the safety in real-life settings.

### 4.2. Efficacy and Safety of Dupilumab 

This study highlighted the remarkable efficacy of dupilumab in managing AD within the first 16 weeks of treatment. In our cohort treated with dupilumab alongside medium-potency TCs, we noted a significant improvement in skin lesions. The EASI score decreased by approximately 78%, and 60% of patients exhibited the complete or near-complete resolution of the affected skin areas. Notably, no extensions of the lichenification processes were observed, aligning closely with the literature findings. The improvement in EASI scores among adults and adolescents (>12 years) has been extensively evaluated in various RCTs [47,48,49], and real-life studies have consistently supported these results [50,51]. A recent multicenter observational real-life study involving 139 adolescents (12–18 years old) displayed a substantial 79.8% reduction in EASI scores after 16 weeks of dupilumab treatment [52]. A significant milestone in recent years was the approval of dupilumab for children aged > 6 years. Its effectiveness in this age group was first assessed by Ingelman et al. [53] in a multicenter retrospective study, which included 124 children with moderate-to-severe AD receiving an off-label treatment. Subsequent studies, including a randomized placebo-controlled phase 3 trial, demonstrated a notable reduction in EASI scores with dupilumab plus TCs compared to placebo plus TCs [54]. Real-life settings also reflected an improvement in skin lesions; Napolitano et al. [55] found that around 75% of 55 children aged 6–11 years achieved a >75% improvement in EASI after 16 weeks. Additionally, a randomized placebo-controlled phase 3 trial conducted in children aged < 6 years exhibited an enhancement in atopic dermatitis signs and symptoms alongside a significant reduction in EASI scores [56]. The secondary parameters investigated were iNRS and sNRS and the quality of life through DLQI/CDLQI score. A reduction in iNRS and sNRS of 31.58% and 46.87%, respectively, was observed. The entire sample of patients reported a decrease in DLQI by at least 4 points, with a mean reduction of 53.93% in the absolute score (*p* = 0.003). No instances of symptom worsening were reported. It is noteworthy that itch and sleep issues notably impact children’s QoL, so improvements or resolutions in these areas signify a better overall quality of life [52]. Larger real-life studies have also shown substantial enhancements in these parameters after 16 weeks of treatment [52,54]. Napolitano et al. [56] reported significant mean percentage reductions in iNRS, sNRS, and c-DLQI (68.39%, 70.22%, and 79.03%, respectively) as early as 2 weeks post-administration, suggesting the drug’s rapid action in children. These benefits persisted in subsequent weeks, markedly enhancing quality of life [55]. A recent multicenter real-world study that spanned 52 weeks and enrolled children aged 6–11 years demonstrated significant improvements in EASI score, iNRS, sNRS, and c-DLQI from baseline to weeks 16, 24, and 52 [57]. Concerning the safety profile, we registered a limited number of AEs, none of which were severe. Erythema on the injection site (*n* = 1) and respiratory tract infections (*n* = 2) were observed, although the association between the latter and drug administration remains uncertain. However, the rate of AEs appeared lower than expected based on the literature data, likely due to the small sample size and the short-term follow-up period. Common AEs reported by manufacturers included conjunctivitis and injection site reactions [8]. This aligns with findings from several RCTs, in which the dupilumab group showed a higher incidence of conjunctivitis and injection site reactions compared to the placebo group [47,48,49]. In a long-term real-world study involving 128 patients (78 adults and 50 children) treated with dupilumab for at least 2 months (mean duration 14.90 ± 10.39 months), the most encountered AEs included head and neck dermatitis (19.5%), conjunctivitis (15.6%), erythema, pruritus, and skin peeling (10.9%), and dryness of the eyes (7.8%) [58]. Similarly, in an Italian multicenter experience, AEs were reported in 14.8% of cases, with conjunctivitis (8.3%) and injection site reactions (6.25%) being the most frequent. No serious AEs were recorded, and none of the children discontinued treatment [57]. Overall, dupilumab appeared safe and well-tolerated, with most AEs being mild and not necessitating drug discontinuation [58]. However, further studies, particularly in children, are necessary to confirm the above reported safety findings.

### 4.3. Limitation of the Study

Our study was predominantly conducted during the peak of the COVID pandemic, making certain functional parameters, such as spirometry, unfeasible due to pandemic-related restrictions. In our country, this diagnostic method was deemed a biological risk, and it was not performed for a long period. Despite the limited sample size, our findings highlighted the significance of the early initiation of biologic treatment when needed for children with severe allergic diseases. While not conclusively demonstrated, it is noteworthy that delaying the onset of biologic treatment could potentially lead to the occurrence of complications, such as prolonged and/or persistent damage to the airways [59].

## 5. Conclusions

Our study confirmed the findings from the existing literature regarding the efficacy, safety, and quality-of-life improvements associated with omalizumab and dupilumab in managing pediatric allergic diseases in real-life settings for the initial 16 weeks of therapy. These preliminary results emphasize the need of starting biologic treatments for children with severe allergic diseases promptly, considering the very low rates of AEs and severe exacerbations leading to drop-out. Real-world studies with longer-term follow-ups and larger sample sizes are needed to better determine the efficacy and safety of biologics in children.

## Figures and Tables

**Table 1:** Demographic and clinical characteristics of study population.

Characteristic	Asthma	CSU	AD	Overall
Number, no. (%)	6 (33.3)	7 (39)	5 (27.7)	18 (100)
Age (years), mean (SD)	10.5 (±3.1)	13.9 (±2.2)	14.4 (±3.3)	12.89 (±3.41)
Male, no. (%)	5 (83.3)	3 (42.9)	3 (60)	11 (61)
BMI (kg/m^2^), mean (SD)	21.1 (±4.0)	27.5 (±4.9)	24.6 (±3.1)	24.57 (±5.13)
Other comorbidities, no. (%)				
Allergic Rhinitis	5 (83.3)	2 (28.6)	5 (100)	12 (66.7)
Allergic Conjunctivitis	4 (66.7)	2 (28.6)	4 (80)	10 (55.5)
Other therapies, no. (%)				
Inhaled corticosteroids	6 (100)	/	/	6 (33.3)
SABA	6 (100)	/	/	6 (33.3)
Oral Corticosteroids	2 (33.3)	1 (14.3)	2 (40)	5 (27.8)
Antihistamines	2 (33.3)	7 (100)	3 (60)	12 (66.7)
Topical corticosteroids	/	/	5 (100)	5 (27.8)
Calcineurin inhibitors	/	/	3 (60)	3 (16.7)

AD: Atopic dermatitis, BMI: body mass index, CSU: Chronic spontaneous urticaria, SABA: short-acting bronchodilators.

## Data Availability

Data are contained within the article.
